# Relational continuity with primary and secondary care doctors: a qualitative study of perceptions of users of the Catalan national health system

**DOI:** 10.1186/s12913-018-3042-9

**Published:** 2018-04-10

**Authors:** Sina Waibel, Ingrid Vargas, Jordi Coderch, María-Luisa Vázquez

**Affiliations:** 1 Health Policy and Health Services Research Group, Health Policy Research Unit, Consortium for Health Care and Social Services of Catalonia, Av. Tibidabo 21, 08022 Barcelona, Spain; 2 Department of Paediatrics, Obstetrics, Gynaecology and Preventive Medicine, Universitat Autònoma de Barcelona, Av. de Can Domènech 737, 08193 Bellaterra (Cerdanyola de Vallès), Spain; 3 Grup de Recerca en Serveis Sanitaris i Resultats en Salut, Serveis de Salut Integrats Baix Empordà, Carrer Hospital 17-19 Edifici Fleming, 17230 Palamós, Spain

**Keywords:** Continuity of patient care, Patient-doctor relationship, Primary health care, Secondary health care, Qualitative research, Patient perspective, Trust, Spain

## Abstract

**Background:**

In the current context of increasingly fragmented healthcare systems where patients are seen by multiple doctors in different settings, patients’ relational continuity with one doctor is regaining relevance; however little is known about relational continuity with specialists. The aim of this study is to explore perceptions of relational continuity with primary care and secondary care doctors, its influencing factors and consequences from the viewpoint of users of the Catalan national health system (Spain).

**Methods:**

We conducted a descriptive-interpretative qualitative study using a two-stage theoretical sample; (i) contexts: three healthcare areas in the Catalan national health system with differing characteristics; (ii) informants: users 18 years or older attended to at both care levels. Sample size (*n* = 49) was reached by saturation. Data were collected by individual semi-structured interviews, which were audio recorded and transcribed. A thematic content analysis was carried out segmenting data by study area, and leaving room for new categories to emerge from the data.

**Results:**

Patients across the areas studied generally experienced consistency of primary care doctors (PCD), alongside some inconsistency of specialists. Consistency of specialists did not seem to be relevant to some patients when their clinical information was shared and used. Patients who experienced consistency and frequent visits with the same PCD or specialist described and valued having established an ongoing relationship characterised by personal trust and mutual accumulated knowledge. Identified consequences were diverse and included, for example, facilitated diagnosis or improved patient-doctor communication. The ascription to a PCD, a health system-related factor, facilitated relational continuity with the PCD, whereas organizational factors (for instance, the size of the primary care centre) favoured consistency of PCD and specialists. Doctor-related factors (for example, high technical competence or commitment to patient care) particulary fostered the development of an ongoing relationship.

**Conclusions:**

Consistency of doctors differs depending on the care level as does the relevance attributed to it. Most influencing factors can be applied to both care levels and might be addressed by healthcare managers to foster relational continuity. More research is needed to fully understand the relevance patients assign to relational continuity with specialists.

**Electronic supplementary material:**

The online version of this article (10.1186/s12913-018-3042-9) contains supplementary material, which is available to authorized users.

## Background

Relational continuity with one doctor is regaining relevance in the current context of increasingly fragmented and depersonalised healthcare systems [1, 2]. In particular patients suffering from pluri-pathologies and chronic conditions are exposed to a higher risk of receiving fragmented care since they are seen by different professionals in various settings [3]. This exposes them to negative consequences of reduced quality of care and potential health hazards if the care delivered is not sufficiently coordinated [4]. A single trusted clinician can help patients to manage their condition and navigate them through the system [5, 6]. In health systems based on primary health care, this is typically the primary care doctor (PCD), however, patients might have consistent contact with a different professional depending on the intensity and type of the care needed [7–9]. Little is known about how perceived elements describing relational continuity might differ between primary and secondary care doctors.

Relational continuity is a complex phenomenon with differing definitions and meanings [10, 11], and conceptual frameworks that can be applied to its analysis continue to be rare [11]. Reid et al. [3] classified relational continuity as one of three types of continuity of care; next to continuity of clinical management (the patient receiving the different services in a consistent way and being responsive to his or her changing needs) and continuity of information (the different providers sharing and using the information on the patient’s past events and personal circumstances). Relational continuity can be defined as an ongoing therapeutic relationship with one or more providers spanning different health care episodes. It is characterised by two dimensions [3, 12]: *consistency of personnel* (also termed longitudinal continuity [13]), which refers to the patient’s perception of being seen by the same professional over time [3]; and an *ongoing therapeutic patient-provider relationship* (also called personal continuity [13], interpersonal continuity [14] or depth of the relationship [11]), which is the patient’s perception of an established relationship with the professional based on trust, mutual understanding and a sense of affiliation between patients and doctors. The core element *care provided over time* should help to distinguish care continuity from related concepts [12], such as the consultation experience [11].

The patient-PCD relationship has been subject to analysis for many decades; arguably since Starfield [15] identified consistency of personnel to be one of the main features of quality primary health care in the early 1990s (together with first-contact, comprehensive and coordinated care across levels). However, only few qualitative studies to date have aimed to understand the full complexity of relational continuity in both of its dimensions and from the viewpoint of patients. Furthermore, important shortcomings in research exist when referring to relational continuity with specialists; a concept which might be defined by different elements to those that constitute relational continuity with PCDs. With reference to the factors influencing relational continuity, studies typically focused on analysing patient-related factors, suggesting that patients with poor self-rated health tend to report lower levels [16], as well as younger [17–20] and foreign-born patients [17, 21, 22]. The identification of other types of influencing factors, such as organizational or doctor-related factors, as perceived by patients, was targeted in some qualitative studies [6, 23, 24], however with the focus set on primary care only.

In the Spanish national health system (NHS), healthcare provision is organized into two levels of complexity: *primary care*, acting as the gatekeeper and being responsible for coordinating the patient’s care along the care continuum, and *secondary care,* acting as a consultant for primary care and being responsible for more complex care [25]. In Catalonia, a multiple-case study with patients with chronic obstructive pulmonary disease (COPD) treated in integrated health care networks [26] and a population survey applying the CCAENA© questionnaire [18, 27] analysed relational continuity together with the other two continuity types, postulating the need for further in-depth analysis [27]. Other Catalan surveys investigated only specific aspects of relational continuity, particularly with PCDs [28, 29].

The aim of this study is to explore perceptions of relational continuity with primary care and secondary care doctors, its influencing factors and consequences from the viewpoint of users of the Catalan NHS. This article presents partial results of a wider study that analyses the relationship between the healthcare services’ perspective of coordination across care levels [30, 31] and the users’ perspective of continuity of clinical management and information across levels of care [32], as well as relational continuity in primary and secondary care (herein presented).

## Methods

### Study design

We carried out a descriptive-interpretative qualitative study with a phenomenological approach, which concentrates on exploring how individuals make sense of the world in terms of the meanings and classifications they employ [33]. In this study, the phenomenon of relational continuity was targeted to be investigated in depth, oriented by Reid et al.’s conceptual framework [3].

### Study sample

We used a theoretical (or criterion) sample because the selection of the informants was based on defined criteria stemming from a literature review and our previous research results [6, 26]. Kuper and colleagues [34] define theoretical sampling as "sampling individuals or texts whom the researchers predict (based on theoretical models or previous research) would add new perspectives to those already represented in the sample". Theoretical or theory based sampling is considered to be a specific strategy of purposeful sampling [35, 36].

The sampling process consisted of two stages. In the first stage, the study contexts were chosen, that represented various management models of primary and secondary care in the NHS of Catalonia as well as different geographical distance to the health facilities. The final selected healthcare areas were: one rural and semi-urban area (Baix Empordà county) and two urban areas (City of Girona, Ciutat Vella of Barcelona). Their characteristics are described in more detail elsewhere [32]. In the second stage, the informants were selected in each context based on the following criteria: healthcare user ≥18 years of age who had been attended to in primary and secondary care (embracing outpatient, inpatient and emergency care) for the same health problem within the three months prior to data collection. We included different variation criteria (sex, age, country of origin and use of different services) in order to capture a broad set of opinions (maximum-variation sampling [34]). Informants were selected by the first author from a list of users provided by the participating organizations. We recruited one respondent through a snowballing technique [34], that means she was recommended by another study participant because of having gathered relevant experience for this study, and responded to the criteria previously defined.

In total, 49 users participated in the study (Table 1). Thirteen users were born outside of Spain, mainly in Latin American countries (*n* = 7); whilst the other study participants were born in North Africa, other European countries and Asia. Users of all socio-economic statuses (from unskilled workers to non-manual and high-level professionals) as well as unemployed and retirees were represented in the study. Most of the unemployed (*n* = 10) were foreign-born and sought care in Ciutat Vella, Barcelona.

**Table 1:** Characteristics of the sample

	Baix Empordà county (*n* = 18)	City of Girona (*n* = 14)	Ciutat Vella (Barcelona) (*n* = 17)	Total (*n* = 49)
Female	9	8	10	27
Age	33–82	22–82	26–70	22–82
Foreign-born	4	4	5	13
Non-native Spanish speaker	2	2	2	6

### Data collection

Individual semi-structured interviews were carried out. The topic guide consisted of a general part about the user’s health and healthcare experiences and a specific part to explore in depth perceptions of the three continuity of care types [3, 12], including relational continuity, which is the focus of this study. The use of a topic guide ensured that all topics of interest were covered during the interview. The final version is available as an annexe in a recent publication that presents the results pertaining to continuity of clinical management and information across the primary and secondary care levels [32].

The detailed data collection process is reported elsewhere [32]. Users were informed that participation was entirely voluntary and would not affect the care they received in any way, and that withdrawal was possible at any point. Consent was obtained from each participant before the interview. In a few cases, carers (who were a family member of the study participant; either his or her spouse or child) joined the interview and provided opinions on the topic of interest. We included this data in the analysis but did not present them separately given that the carers’ opinions did not differ from but corroborated the patients’ narrative. All interviews were audio recorded and transcribed in full. To enhance reflexivity [34], field notes were taken in the different steps of the research process and discussed with members of the research team when necessary. Data saturation [34] was achieved in each of the three study areas.

### Data analysis

A thematic content analysis was carried out by the first author, with data managed using the Atlas-ti 5.0 software. We segmented data by study area to analyse the phenomenon of relational continuity in the different healthcare contexts. We further conducted a mixed generation of categories; that means, we left room for new categories to emerge apart from those represented in the topic guides [37]. We coded the transcripts and developed and refined the categories while examining new sections of text. Three researchers who were knowledgeable about qualitative research, the study context and phenomenon triangulated the data to enhance quality of findings.

## Results

**Fig. 1:**
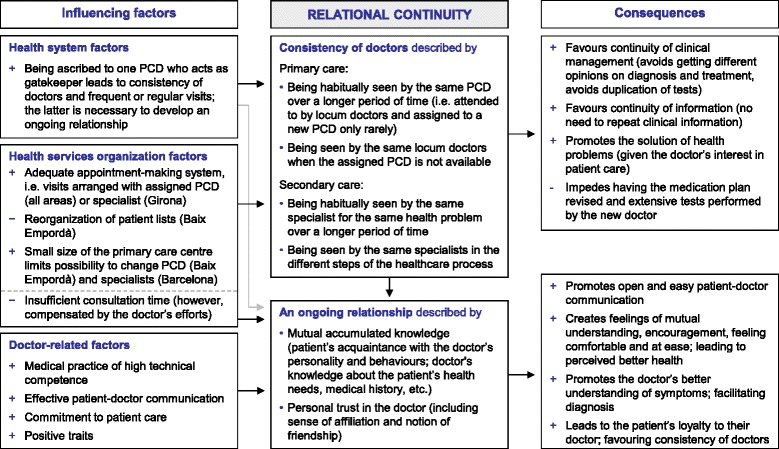
Influencing factors, definition and consequences of relational continuity with primary and secondary care doctors

From the discourse emerged that patients across the three healthcare areas studied generally experienced consistency of PCDs while perceptions of consistency of specialists along the care process varied. Those patients who experienced frequent visits over time with the same primary care or secondary care doctor indicated that they were able to establish an ongoing relationship. Identified consequences were diverse and included, for example, facilitated diagnosis, no duplication of tests or improved patient-doctor communication. Relational continuity was linked to influencing factors related to the health system, health services organizations and doctors. The results of the patients’ perceptions of relational continuity in both care levels, its influencing factors and consequences are summarized in Fig. 1. Additional file 1 provides further quotations to those presented hereafter, exemplifying our results. Additional file 2 contains the original Spanish language version of all selected quotations.

### Being seen by the same doctor over time more common and relevant in primary care

In primary care, patients in all healthcare areas generally experienced and highly appreciated that there was consistency of doctors, which was defined as habitually being seen by their assigned PCD and only rarely by different locum doctors when there was a specific reason, such as the PCD being on holiday, maternity or sick leave: *Locum doctors? Not that I’ve seen. (...) At the health centre, I nearly always see my doctor (...) unless, of course, she’s off work... (Barcelona, male patient).* During their doctor’s absence, some patients indicated and appreciated that they were seen by the same locum doctor. Moreover, patients highlighted that they were ascribed to the same doctor over a longer period of time (from a couple of years to over fifteen years), which only changed given a warrantable reason, such as the doctor’s retirement or the patient’s change of residence.

With reference to secondary care doctors, the patients’ perceptions of consistency varied; some patients across the healthcare areas pointed out that they were followed up by the same specialist over time, whilst others indicated inconsistency, described as being seen for the same health problem by a different doctor of the same speciality, or in the different steps of the healthcare process, including pre-operative and follow-up visits, when undergoing surgery. To maintain consistency, some patients paid out of pocket to receive follow-up care from the same surgeon that had also performed the operation. Nevertheless, numerous patients did not consider it important to be seen by the same specialist for every medical consultation because they found that all specialists were competent enough to treat their health problem and that they usually shared and used their clinical information, which facilitated the change: *Sometimes you have an appointment with one doctor but for some reason, he can’t on this day (…) that doesn’t matter, on the contrary, the new one was well informed about what had happened to me, he knew perfectly. I didn’t notice the difference (…) he had read my medical history (Baix Empordà, female patient).*

Being seen by different PCDs was linked to a number of negative consequences: receiving inconsistent opinions on diagnosis and treatment, not having the health problem solved (attributed to the locum doctor’s lack of interest in patient care), having to repeat clinical antecedents (because the locum doctor lacked time to read the clinical history or specific information was inaccessible to them) and in some cases the patient seeking private health care. Duplication of medical tests and the need to repeat their clinical history were mentioned as negative consequences of being seen by different specialists. However, some patients saw positive implications in being seen by new specialists because they performed extensive tests and revised and adapted their medication plan: *(The new neurologist) is great. (…) She changed my medicine. She told me I’d been taking the migraine medicine too long and that it could do damage to my heart, and my kidneys and my eyesight as well. She changed it and, well, now I’m trying a new one so let’s see how I bear up (Barcelona, male patient, 48).*

### An ongoing relationship with primary and secondary care doctors based on mutual accumulated knowledge and personal trust

An ongoing relationship (with the PCD, specialist or locum doctor) was characterised by two main features. First, patients described mutual accumulated knowledge, which goes beyond recognizing each other (*putting a name to a face*), and was developed gradually by the patients getting acquainted with the doctor’s personality, social contexts and *the way the doctor behaved and reacted*; and the doctor acquiring knowledge about the patient’s health needs, character, social circumstances and medical history: *I’ve been with her (the General Practitioner - GP) almost 10 years (…) the doctor knows you and hardly has any need to look at your records because he or she knows all about it (referring to the diagnosis made and treatment prescribed) (Barcelona, male patient)*. Second, patients described personal trust in their doctor built over time, in addition to the trust shown in the doctor’s technical competence. The latter was developed based on the patient’s positive or absent negative experience of the primary and secondary care received and was considered, by the patient, to be fundamental to achieve adherence to the treatment plan.

Patients across the areas generally indicated and valued that they had developed an ongoing relationship with their PCD because they had been seeing them over a longer period of time as well as on a frequent or regular basis. Some patients further showed a sense of affiliation by talking about *my doctor*, and others highlighted that their acquaintance even turned into a friendship and a feeling of the PCD *belonging to the family*. Some patients who had developed personal trust pointed out that they more readily "accepted" the PCD’s medical errors or lesser technical competence. With reference to secondary care, many patients did not develop an ongoing relationship due to experiencing inconsistency of specialists and a lack of periodic visits, in contrast to the relationship established with their PCD: *Well of course I can’t talk about a specialist like I can about my GP, because I only go to the specialist every now and then. With my GP we deal with every little thing, illnesses, colds, this and that, I see him a lot (…) the relationship is closer (Girona, male patient).* The few patients who were followed up by the same specialist over time reported that they had established a strong bond.

Patients highlighted that an ongoing relationship led to open and easy conversations based on mutual understanding, encouragement, and feeling comfortable and at ease, which in turn was perceived to result in better health. Patients further indicated that their doctor understood their symptoms better facilitating diagnosis. A developed bond also led to patient loyalty toward their primary or secondary care doctor, which became apparent through their actions, such as continuing to see the same PCD after having changed address or waiting longer to be seen by their own PCD: *You can have confidence in anyone but if she’s the one who’s always seen you, she’s your doctor, the doctor you trust. I’ve seen a lot of people at the clinic just get up and leave because they saw that this particular doctor wasn’t there (Baix Empordà, female patient).*

### Factors related to the health system

Patients across the study areas pointed out that being assigned to one PCD who acts as gatekeeper (*having to consult the PCD initially for any health problem*) led to consistency as well as frequent or periodic visits with the same PCD, which was seen to be fundamental to developing an ongoing relationship. This was further facilitated by the patient sharing the PCD with other family members, as was the case with a few patients: *Maybe I’ll go with someone from the family, I’ll accompany them, I see her a lot (…) the relationship is closer (Girona, male patient).*

### Factors related to health services organizations

Patients across the areas related consistency of PCDs to the organization’s appointment-making system, given that non-urgent visits were usually arranged with their PCD, according to his or her availability. This was also the case for secondary care in Girona, where patients reported that visits were postponed when the specialist from the initial appointment was not available, which equally favoured consistency: *The specialist for the digestive system (…) has always been the same one, a gastroenterologist (…) because if that appointment is with her and she can’t make it then they tell me and they give me another date, but it’s always the same one (Girona, female patient).*

A few patients from Baix Empordà related inconsistency of primary or secondary care doctors to a re-organization of patient lists. This, patients highlighted, was needed to better distribute the workload between doctors, which they associated with high immigration in the area: *(…) looks like they were doing a bit of organising with the doctors and patients. I suppose it’s probably because there are a lot of immigrants, Moroccans who don’t feel comfortable with their doctors (…) well as the doctor had too many clients, or rather patients, I suppose they had to do a bit of reorganising (Baix Empordà, male patient).*

According to a few patients, having a small primary care centre limited the possibility of changing PCDs (in Baix Empordà county) and specialists (who offered visits in primary care centres in Barcelona), thus indirectly favouring consistency of personnel: *I’ve never considered it (changing the GP), to be honest, I’ve never thought about it because over in Calonge I think they’ve only got two doctors. There are two doctors, it would just be changing from one to the other (Baix Empordà, male patient).*

Insufficient consultation time in both care levels was generally experienced by patients across the areas to detract from developing an ongoing patient-doctor relationship, affecting patient-doctor communication and the doctor’s ability to provide personalized or tailored care. A few patients linked not having enough consultation time to a general shortage of personnel and resources in both care levels; whereas having sufficient time in secondary care was related to a smaller size of the hospital: *In this case here at Hospital del Mar (…) well I might spend half an hour talking about it (with the neurologist and nephrologist). At least they seem to care more about their patients. At the Hospital Clínico, they file them through, so to speak. You go in, bla bla bla, done, out. I guess it’s because as it’s bigger, it’s more centralized, as they’ve got a lot more people to deal with (Barcelona, male patient).*

### Factors related to doctors

A medical practice of high technical competence fostered trust in primary and secondary care doctors and thus facilitated the development of an ongoing relationship. In primary care, good practice was described by patients as making the right diagnosis, prescribing the treatment they needed, referring them to secondary care when necessary and performing accurate examinations and all medical tests needed: *My GP, what can I say, she’s fantastic, she’s a wonderful person (…) because she’s a lady who pays a lot of attention to everything, she monitors you constantly (...) she does tests, she examines me to see if there’s anything wrong, she checks my prostate, she checks this, she checks... everything (Girona, male patient).* The specialist’s high technical competence was mainly linked to precise revision of the medication plan, accurate physical examinations and making referrals to a different specialist when necessary to get the diagnosis confirmed.

Effective patient-doctor communication enhanced the development of trust and mutual understanding with both primary and secondary care doctors. To patients, it meant not only receiving detailed explanations of their condition, treatment options and side effects in an appropriate manner (friendly, tactful and calm), but also the chance to talk without time pressure about their health and upcoming visits to different care levels, as well as other unrelated topics: *Last time we went, we chatted for a bit, but we were talking about the cutbacks and stuff (…) The doctor-patient barrier, which is significant and all that, well that was lowered a bit. She became more approachable and I suppose I also became more approachable to her; it was a more human relationship, shall we say (Barcelona, male patient).* For some patients, sharing similar demographic characteristics with their doctors (age, sex) made them feel at ease and thus enhanced open and honest conversations.

The doctor’s commitment to patient care was perceived to promote the creation of a trusting relationship, particularly with the PCD, by providing personalized care and preventing the patient from ‘feeling like a number’: *I have quite a lot of trust in her (GP), yes. And I think that she makes an effort (…) she cares about the patient, and I think that’s important (Barcelona, female patient).* Commitment included taking the health problem seriously, showing interest in the patient’s wellbeing and demonstrating dedication to their work. It was also described through their PCD’s actions: calling patients to ask after their wellbeing, reminding them of annual check-ups, making home visits, reading their clinical history before the visit and being accessible by phone even out of working hours. The PCDs’ commitment to work was perceived to depend on their age: young doctors were more motivated, whilst some elder doctors seemed to be burned-out after having worked for many years in the field.

Finally, a number of positive traits among doctors of both care levels enhanced the development of a bond, such as the doctor having a kind, cheerful, open, attentive, tactful and calm character: *(My GP) is friendly, very good-natured, very calm. He’s nice. You feel you can trust him (Girona, female patient).*

## Discussion

There are few qualitative studies to date that aim to understand the full complexity of relational continuity in both of its dimensions (consistency of personnel and an ongoing relationship [3]), particularly in reference to secondary care. This study contributes to enhancing our knowledge by exploring relational continuity with primary and secondary care doctors, identifying the different types of factors influencing it as well as its consequences, as perceived by patients attended to in different healthcare areas of the Catalan NHS.

### Consistency of primary care doctors promoted by the primary care model and organizational factors

Patients described consistency of PCD as usually being seen by the same doctor over an extended period of time and by locum doctors only on rare occasions. They generally confirmed that consistency existed; a result consistent with those of a cross-sectional user survey conducted in the same study areas, where 80% reported that they were seen by the same PCD throughout the previous year [27]. The PCD’s gatekeeper function seems to enhance not only experienced consistency of PCD but also the development of an ongoing relationship when patients had frequent or periodic visits in primary care. Thus, our results appear to indicate that a healthcare model based on primary health care, as promoted by the Spanish NHS, generally fosters relational continuity with the PCD. In contrast, the specialist acting as the technical expert and consultant for primary care, presumably does not favour relational continuity to the same extent or even distracts from it, possibly explaining why numerous patients experienced inconsistency.

Our study also shows that, apart from having an assigned PCD, different organizational factors needed to coexist to guarantee consistency. One important factor appeared to be the appointment-making system implemented in the areas, which determined if visits were finally arranged with the patient’s PCD [38]. Other studies identified that offering scheduled visits in the evenings [39] and having short waiting times for the preferred PCD [40] favoured continuity, and that the receptionist also had an important role to play in maintaining consistency [24]. Furthermore, consistent with previous work by Guthrie [41], the small size of primary care centres was identified to be a factor that favoured consistency. Our results suggest that small centres limited the possibility of changing PCDs, even if the patient wished to do so. Furthermore, we hypothesize that PCDs practicing in rural areas are more loyal to their workplace, thus there is less rotation. In contrast to these results, patients reported lower continuity in rural areas in Canada, possibly because these areas were underserved by doctors [20].

### Different potential rationales for the different value attached to consistency of specialists

Patients across the areas highlighted inconsistency of specialists, described as being seen by a different doctor of the same speciality on every visit, rather than the same one throughout the care process. In the user survey, however, 85% reported that they were seen by the same specialist in the previous year for the same condition (ranging from 87% in Barcelona to 81% in Baix Empordà) [27]. Our qualitative results might indicate that patients refer to a longer time span when talking about consistency, which thus could determine their response in surveys.

The results also revealed that, interestingly, being seen by the same specialist did not seem to be relevant to some patients when their clinical information was shared with and taken up by the new doctor, and when the specialist was considered competent enough to treat their health problem [38]. Four possible rationales could serve to explain these findings. First, speed of access to specialist appointments might be prioritized over relational continuity by patients with specific socio-demographic characteristics. The patient patterns might be similar to those who value receiving a convenient appointment time with any doctor more than being seen by their own PCD [42–44]. Second, seeing a different doctor offers other benefits, such as allowing the patient to seek a second opinion [23, 45], having the treatment plan revised and new tests performed (a result from our study), or receiving better care because the doctor might possess greater technical skills [23]. Third, patients lacked shared experiences with the specialist because of the observed inconsistency of doctors, and thus valued continuity less; which was shown elsewhere with regards to PCDs [46]. And fourth, patients wish to have a single trusted doctor who takes on the responsibility of coordinating their care and providing follow-up, which is typically the PCD in the NHS; however, patients with chronic conditions might prefer consistent contact with the specialist, as became apparent in a Danish qualitative study with COPD patients [8], as well as in a Swedish quantitative study on breast cancer [9].

### How can an ongoing relationship based on trust and accumulated knowledge be achieved?

According to our results, three major components – that seem to be applicable to both primary and secondary care doctors – need to coexist to be able to establish an ongoing relationship, in which patient and doctor know each other and the patient’s personal trust in the doctor adds to his or her general trust in the doctor’s technical competence. *Consistency of personnel* is perhaps the most important component and shows the interdependence between the two dimensions of relational continuity, which was also described in a meta-synthesis of qualitative studies [6] and a quantitative study with US Medicare patients [47]. *Frequency (or intensity) of visits* is the second essential component, highlighted previously in a qualitative study with type 2 diabetes patients [48] and in cross-sectional studies conducted in the US [49] and the English NHS [40]. Patients in our study linked the gatekeeper system with increased primary care visits, which in turn facilitated the development of an ongoing relationship with the PCD. As with consistency, frequency of visits indicates ‘whether there has been sufficient opportunity for the patient to develop a relationship with the doctor’ [39]. Previous research suggests that consistency and frequency are not independently associated with the development of trust in a doctor [17], but a third component has to be present to develop an ongoing relationship: *the patient’s consultation experience* or the quality of the encounter, which has a major bearing on how the relationship develops [11]. In our study, a combination of four intertwined doctor-related factors defines the consultation experience: technical competence, effective patient-doctor communication, the doctor’s commitment to patient care and positive traits. All factors emerged in relation to both the primary and secondary care doctor, however specific elements describing these factors were related to one care level only, for example making the right diagnosis or referring the patient to secondary care, which described the quality of PCD visits.

### Potential of relational continuity to improve quality of health care delivery

A number of consequences of relational continuity with both the primary and secondary care doctor were identified. All of them had positive connotations and were mainly related to the quality of care received, and only to a very limited degree to the patients’ health. Numerous quantitative studies analysed the associations between consistency of doctors and different outcomes, showing that consistency was frequently associated with decreased health care utilization (including hospitalization and emergency visits) and increased patient satisfaction [50, 51]; however it remains unclear if consistency leads to better clinical outcomes [51].

Our results add additional consequences of relational continuity with primary and secondary care doctors to the existing literature such as facilitated diagnosis or solution of health problems given the doctor’s interest in patient care. Moreover, relational continuity seems to enhance continuity of clinical management and information, for instance, by avoiding duplication of tests or the need to repeat clinical information to the new doctor. Thus our results support the concept of the interrelation of the three continuity types [6, 26].

Another relevant consequence of an ongoing relationship is the efforts that patients make to continue with the same doctor, also called loyalty, which has been described in other frameworks as an element of the strength or depth of the doctor-patient relationship [11, 14]. Loyalty was expressed in primary care, for instance, by the patient following the PCD to a different primary care centre, and in secondary care, by the patient paying out-of-pocket to receive follow-up care with the same specialist who performed the surgery in the public system. Literature also highlights that patients might disagree on being handed over to secondary care and are willing to sacrifice clinical expertise of specialists for the security of being looked after by their known PCD [5]. Finally, it should be noted that loyalty, in turn, favours consistency of doctors, and therefore acts as an influencing factor, demonstrating the full complexity of the phenomenon.

### Study limitations

One main study limitation warrants consideration. The information collected on patients’ experiences with a developed ongoing relationship with specialists, the second dimension, was limited. This is due to the inclusion criteria – patients who were attended to in both primary and secondary care for the same reason within the three months prior to the interview – which was initially designed to also being able to analyse continuity of clinical management and information across care levels [32]. Thus, patients who had only one or a few specialist visits were included in the study. In addition, a number of patients experienced inconsistency of specialists, thus further reducing the amount of information obtained on the concept of an ongoing relationship. Given the intertwined nature of both dimensions, any study investigating the phenomena would encounter the same limitation, demonstrating its complexity.

### Recommendations for healthcare organizations and future research

Healthcare managers should aim to maintain and improve experienced relational continuity with PCDs by providing patients with sufficient opportunities to develop an ongoing relationship, given that relational continuity shows potential to improve quality of care (including continuity of clinical management and information) and subsequently, in all likelihood, the patient’s health. Furthermore, some types of patients may consider it relevant to be seen by the same specialist over a longer period of time, however this has to be confirmed in future research. To achieve relational continuity in primary care, managers might first target consistency by addressing the different organizational factors identified (for example, improving the appointment-making system or reconsidering the need to change patient lists) and second, facilitate the development of an ongoing relationship by guaranteeing that doctors are able to dedicate enough time to patients during visits (for example, reducing the number of patients per doctor). Health professionals in turn could support the development of a trusting relationship with patients by means of developing communication and consultation skills, aiming to adopt good medical practice tailored to the patient’s individual needs, and demonstrating sufficient interest in patient care [6, 26].

Three recommendations are given for future research. This study provides a first approach for describing relational continuity with the specialist. Further qualitative studies are needed to better understand patients’ perceptions and needs regarding relational continuity with specialists in different contexts. Aiming to comprehend the linkages between relational continuity and quality of care should warrant more in-depth consideration in the future, given that continuity of care is already purported to be a critical feature of high quality services, albeit with still limited evidence [52]. Finally, future studies using quantitative methods might aim to analyse the relative significance of the different elements explaining relational continuity, and also attempt to demonstrate causal relationships between the factors that seem to influence relational continuity as well as the potential consequences identified here.

## Conclusions

This study suggests that patients’ experiences of relational continuity are similar in the selected healthcare areas of the Catalan NHS, where consistency of PCDs is considered to exist alongside some inconsistency of specialists. Consistency of doctors and frequent visits with the primary and secondary care doctor appear to be a prerequisite to establishing an ongoing relationship characterised by mutual accumulated knowledge and personal trust. The different influencing factors of relational continuity are related to the health system (ascription to one PCD), health services organization (appointment-making system, reorganization of patient lists, size of the primary care centre, consultation time) and doctors (technical competence, patient-doctor communication, commitment to patient care and doctors’ traits), and seem to be applicable to both care levels, except for the system-related factor, which favours relational continuity in primary care only. Given its positive consequences (including fostering continuity of clinical management and information), healthcare managers and doctors should aim to deliver relational continuity, particularly in primary care. More research is needed to fully understand the phenomena of relational continuity with specialists and the relevance patients assign to it.

## Additional files

Additional file 1: Examples of quotations of relational continuity. (PDF 293 kb)

Additional file 2: Original Spanish version of the quotations. (PDF 291 kb)

## Abbreviations

COPD: Chronic obstructive pulmonary disease
GP: General practitioner
NHS: National health system
PCD: Primary care doctor

## References

[1] Stange K, Burge F, Haggerty JL (2014). RCGP continuity of care toolkit: promoting relational continuity. Br J Gen Pract.

[2] Stange K (2009). The problem of fragmentation and the need for integrative solutions. Ann Fam Med.

[3] Reid R, Haggerty JL, McKendry R (2002). Defusing the confusion: concepts and measures of continuity of healthcare.

[4] Bodenheimer T (2008). Coordinating care--a perilous journey through the health care system. N Engl J Med.

[5] Haggerty JL, Roberge D, Freeman GK, Beaulieu C (2013). Experienced continuity of care when patients see multiple clinicians: a qualitative metasummary. Ann Fam Med.

[6] Waibel S, Henao D, Aller MB, Vargas I, Vázquez ML (2012). What do we know about patients’ perceptions of continuity of care? A meta-synthesis of qualitative studies. Int J Qual Health Care.

[7] Haggerty JL (2012). Ordering the chaos for patients with multimorbidity. BMJ.

[8] Wodskou PM, Høst D, Godtfredsen NS, Frølich A. A qualitative study of integrated care from the perspectives of patients with chronic obstructive pulmonary disease and their relatives. BMC Health Serv Res. 2014;14:471. 10.1186/1472-6963-14-471 PMC4283082 25277208

[9] Bergenmar M, Nylen U, Lidbrink E, Bergh J, Brandberg Y (2006). Improvements in patient satisfaction at an outpatient clinic for patients with breast cancer. Acta Oncol.

[10] Saultz JW, Albedaiwi W (2004). Interpersonal continuity of care and patient satisfaction: a critical review. Ann Fam Med.

[11] Ridd M, Shaw A, Lewis G, Salisbury C (2009). The patient-doctor relationship: a synthesis of the qualitative literature on patients’ perspectives. Br J Gen Pract.

[12] Haggerty JL, Reid RJ, Freeman GK, Starfield BH, Adair CE, McKendry R (2003). Continuity of care: a multidisciplinary review. BMJ.

[13] Freeman G, Hjortdahl P (1997). What future for continuity of care in general practice?. BMJ.

[14] Saultz JW (2003). Defining and measuring interpersonal continuity of care. Ann Fam Med.

[15] Starfield B (1992). Primary care: concept, evaluation, and policy.

[16] Gulliford M, Cowie L, Morgan M (2011). Relational and management continuity survey in patients with multiple long-term conditions. J Health Serv Res Policy.

[17] Tarrant C, Stokes T, Baker R (2003). Factors associated with patients’ trust in their general practitioner: a cross-sectional survey. Br J Gen Pract.

[18] Aller MB, Vargas I, Waibel S, Coderch J, Sánchez-Pérez I, Colomés L, Llopart JR, Ferran M, Vázquez ML (2013). A comprehensive analysis of patients’ perceptions of continuity of care and their associated factors. Int J Qual Health Care.

[19] Schers H, van den Hoogen H, Bor H, Grol R, van den Bosch W (2005). Familiarity with a GP and patients’ evaluations of care. A cross-sectional study. Fam Pract.

[20] Kristjansson E, Hogg W, Dahrouge S, Tuna M, Mayo-Bruinsma L, Gebremichael G (2013). Predictors of relational continuity in primary care: patient, provider and practice factors. BMC Fam Pract.

[21] Mead N, Roland M (2009). Understanding why some ethnic minority patients evaluate medical care more negatively than white patients: a cross sectional analysis of a routine patient survey in English general practices. BMJ.

[22] Aller MB, Colomé JM, Waibel S, Vargas I, Vázquez ML (2013). A first approach to differences in continuity of care perceived by immigrants and natives in the Catalan public healthcare system. Int J Environ Res Public Health.

[23] Parker G, Corden A, Heaton J (2011). Experiences of and influences on continuity of care for service users and carers: synthesis of evidence from a research programme. Health Soc Care Community.

[24] Alazri M, Heywood P, Neal RD, Leese B (2007). Continuity of care: literature review and implications. Sultan Qaboos Univ Med J.

[25] Ley 14/1986. 25 de abril, General de Sanidad. B.O.E. Spain.

[26] Waibel S, Vargas I, Aller MB, Gusmão R, Henao D, Vázquez ML (2015). The performance of integrated health care networks in continuity of care: a qualitative multiple case study of COPD patients. Int J Integr Care.

[27] Aller MB, Vargas I, Waibel S, Coderch J, Sánchez-Pérez I, Llopart JR, Colomés L, Ferran M, Garcia-Subirats I, Vázquez ML (2013). Factors associated to experienced continuity of care between primary and outpatient secondary care in the Catalan public healthcare system. Gac Sanit.

[28] Bartoll X, Salvador M, Allué N, Borrell C. Enquesta de salut de Barcelona 2011. Barcelona: Agència de Salut Pública de Barcelona; 2011.

[29] Generalitat de Catalunya. Departament de Salut. La veu de la ciutadania: com la percepció de la ciutadania es vincula a la millora dels serveis sanitaris i el sistema de salut de Catalunya. Barcelona: Servei Català de la Salut; 2015.

[30] Aller MB, Vargas I, Coderch J, Calero S, Cots F, Abizanda M, Farré J, Llopart JR, Colomés L, Vázquez ML. Development and testing of indicators to measure coordination of clinical information and management across levels of care. BMC Health Serv Res. 2015;15:323. 10.1186/s12913-015-0968-z PMC4535786 26268694

[31] Aller MB, Vargas I, Coderch J, Calero S, Cots F, Abizanda M, Colomés L, Farré J, Vázquez ML. Doctors’ opinions on clinical coordination between primary and secondary care in the Catalan healthcare system. Gac Sanit. 2017; 10.1016/j.gaceta.2017.06.001. 10.1016/j.gaceta.2017.06.001 28844783

[32] Waibel S, Vargas I, Aller MB, Coderch J, Farré J, Vázquez ML (2016). Continuity of clinical management and information across care levels: perceptions of users of different healthcare areas in the Catalan national health system. BMC Health Serv Res.

[33] Reeves S, Albert M, Kuper A, Hodges BD (2008). Why use theories in qualitative research?. BMJ.

[34] Kuper A, Lingard L, Levinson W (2008). Critically appraising qualitative research. BMJ.

[35] Palinkas LA, Horwitz SM, Green CA, Wisdom JP, Duan N, Hoagwood K (2015). Purposeful sampling for qualitative data collection and analysis in mixed method implementation research. Admin Pol Ment Health.

[36] Ayres L (2007). Qualitative research proposals--part III: sampling and data collection. J Wound Ostomy Continence Nurs.

[37] Pope C, Ziebland S, Mays N (2000). Qualitative research in health care: analysing qualitative data. BMJ.

[38] Waibel S (2015). Continuity of health care across care levels in different healthcare areas in the Catalan national health system: the patient’s perspective. Doctoral thesis.

[39] Haggerty JL, Pineault R, Beaulieu MD, Brunelle Y, Gauthier J, Goulet F, Rodrigue J (2008). Practice features associated with patient-reported accessibility, continuity, and coordination of primary health care. Ann Fam Med.

[40] Kearley KE, Freeman GK, Heath A (2001). An exploration of the value of the personal doctor-patient relationship in general practice. Br J Gen Pract.

[41] Guthrie B (2002). Continuity in UK general practice: a multilevel model of patient, doctor and practice factors associated with patients seeing their usual doctor. Fam Pract.

[42] Pandhi N, Saultz JW (2006). Patients' perceptions of interpersonal continuity of care. J Am Board Fam Med.

[43] Nutting PA, Goodwin MA, Flocke SA, Zyzanski SJ, Stange KC (2003). Continuity of primary care: to whom does it matter and when?. Ann Fam Med.

[44] Aboulghate A, Abel G, Elliott MN, Parker RA, Campbell J, Lyratzopoulos G, Roland M (2012). Do English patients want continuity of care, and do they receive it?. Br J Gen Pract.

[45] Mercer SW, Cawston PG, Bikker AP (2007). Quality in general practice consultations; a qualitative study of the views of patients living in an area of high socio-economic deprivation in Scotland. BMC Fam Pract.

[46] Mainous AG, Goodwin MA, Stange KC (2004). Patient-physician shared experiences and value patients place on continuity of care. Ann Fam Med.

[47] Nyweide DJ (2014). Concordance between continuity of care reported by patients and measured from administrative data. Med Care Res Rev.

[48] Alazri M, Neal RD, Heywood P, Leese B (2006). Patients' experiences of continuity in the care of type 2 diabetes: a focus group study in primary care. Br J Gen Pract.

[49] Noyes R, Kukoyi OA, Longley SL, Langbehn DR, Stuart SP (2011). Effects of continuity of care and patient dispositional factors on the physician-patient relationship. Ann Clin Psychiatry.

[50] Health Quality Ontario. Continuity of care to optimize chronic disease management in the community setting: an evidence-based analysis. Ont Health Technol Assess Ser. 2013;13:1–41. PMC3806147 24167540

[51] van Walraven C, Oake N, Jennings A, Forster AJ (2010). The association between continuity of care and outcomes: a systematic and critical review. J Eval Clin Pract.

[52] van Servellen G, Fongwa M, Mockus D’Errico E. Continuity of care and quality care outcomes for people experiencing chronic conditions: a literature review. Nurs Health Sci. 2006;8:185–95. 10.1111/j.1442-2018.2006.00278.x 16911180

