# Correction to: Relational continuity with primary and secondary care doctors: a qualitative study of perceptions of users of the Catalan national health system

**DOI:** 10.1186/s12913-018-3268-6

**Published:** 2018-06-11

**Authors:** Sina Waibel, Ingrid Vargas, Jordi Coderch, María-Luisa Vázquez

**Affiliations:** 1 Health Policy and Health Services Research Group, Health Policy Research Unit, Consortium for Health Care and Social Services of Catalonia, Av. Tibidabo 21, 08022 Barcelona, Spain; 2 Department of Paediatrics, Obstetrics, Gynaecology and Preventive Medicine, Universitat Autònoma de Barcelona, Av. de Can Domènech 737, 08193 Bellaterra (Cerdanyola de Vallès), Spain; 3 Grup de Recerca en Serveis Sanitaris i Resultats en Salut, Serveis de Salut Integrats Baix Empordà, Carrer Hospital 17–19 Edifici Fleming, 17,230, Palamós, Spain

## Correction

Following publication of the original article [1], the authors reported a correction in affiliation of Maria Luisa Vázquez, who is affiliated with Health Policy and Health Services Research Group, which is number 1 instead of being affiliated with Grup de Recerca en Serveis Sanitaris i Resultats en Salut, Serveis de Salut Integrats Baix Empordà, Carrer Hospital 17–19 Edifici Fleming, 17,230 Palamós, Spain.

The affiliation in the original article is:

Sina Waibel ^1,2*^, Ingrid Vargas^1^, Jordi Coderch^3^ and María-Luisa Vázquez^3^.

The correct affiliation is:

Sina Waibel^1,2*^, Ingrid Vargas^1^, Jordi Coderch^3^ and María-Luisa Vázquez^1^.
