# Supplementary material for: Relational continuity with primary and secondary care doctors: a qualitative study of perceptions of users of the Catalan national health system
Source: BMC Health Serv Res. 2018 Apr 10;18:257. doi: 10.1186/s12913-018-3042-9 (PMC5891958; doi:10.1186/s12913-018-3042-9)
Supplement: Supplementary file 1 — Examples of quotations of relational continuity. (PDF 293 kb) [file 12913_2018_3042_MOESM1_ESM.pdf]

Supplementary file 1 – Examples of quotations of relational continuity with primary and secondary care doctors

A. Patients' perceptions of relational continuity

Category	Quotations	
	**Primary care doctor**	**Secondary care doctor**
Consistency of doctors over time	*Since the other one (GP) I had retired, well, I don't know, it must be 15 or 20 years now that I've been with this one. It's been a long, long time (…) Always the same one. (Baix Empordà, male patient)*<br>*Now and again she's had to go someplace, and I've made an appointment with another doctor (…) very occasionally that is (…) just once or twice. (Girona, male patient)*<br>*With this doctor, perfect. Once it turned out that she was on holiday, or once I think she was ill but that's normal, we all get ill, and we all take holidays. (Baix Empordà, female patient)*	*Well sometimes you're given an appointment for a particular day and that day the doctor (specialist) can't make it for whatever reason, and they give you another one, a locum doctor (specialist) (…) it doesn't bother me, no, quite the opposite, he was fully informed on what my problem was, he knew perfectly well and there was no problem, so I couldn't tell the difference (Baix Empordà, male patient)*<br>*The oncologist, she's only been changed on me once (…) Of course, I mean there's staff turnover everywhere, isn't there? So in oncology, yes, there was a change, but I would even see her (the original doctor) in the other office because she changed specialty (…) they seemed very, you know, like, very coordinated doing the transfer (…) I mean, coordinated because one left and the other one just replaced her. "Yes, yes, she told me such and such." (…) it was no problem (Barcelona, female patient).*
Developed ongoing relationship based on		
a) mutual accumulated knowledge	*She knows me; I mean I think my mother knew me well, but she knows me even better. She even said to me once "you're the kind of person who hardly ever complains". And it's true. Sometimes I find it*	*For the lumbago, he saw me two or three times and so yes, he does know me, now of course if I don't go back for another six months, well he might just recognise me, but no, that's normal. If there's a*

	maddening, I say "you know me better than I know myself!" (… The locum doctor) doesn't have that personal knowledge or have it memorized like she does, for example, the way she knows me as a person. (Girona, male patient)<br>*If she (GP) is on holiday or something, well yes, there are locum doctors, of course (…) they just take a quick look and off you go, because it's different with her, you see. The locum doctor doesn't really know what's wrong with you even though it's there on the computer, that this man has high blood sugar or blood pressure or whatever, but it's not the same, is it? My doctor already knows these things without having to look anything up. (Baix Empordà, male patient)*	*period where you go a lot, it's like with everyone, if you see a person a lot you end up getting to know them, but if you only see them now and again, they ring a bell but that's it. (Barcelona, male patient)*<br>*(The orthopaedic surgeon and the GP) are doctors that have been with us for years. Not just four years or eight years. Many, many years. And we know each other well. And they know what we're like and we know more or less what they do. We're happy with them. (Girona, female patient)*
b) personal trust in the doctor	*My GP is the one I trust in the most because it's always been the same one up till now (…) I've been with him for more than 30 years. I prefer to come here and see you (him) (…) like one of the family so I can confide in you (him) and talk openly. (Girona, male patient)*<br>*They don't treat you like they should in emergencies, they just deal with each patient as they come in. (The GP) is better because there's already a degree of trust, isn't there? You feel more free to talk and to tell them about it than with doctors who treat you occasionally, you don't have much of a relationship with them. (Girona, male patient)*	*With the cardiologist (…) I have a close relationship and it's always been him who's attended to me here. He inspires me with confidence and I can see that what he tells me, I understand and I can see that he's right. I have a lot of trust in him, and he's never let me down yet, so yeah, good. (Barcelona, male patient)*<br>*Sometimes, I don't know if it was from stress but (the physiotherapist) was a bit rude. (…) Um, trust…well I'd say no, because if there was trust, I think I would have continued with the treatment until she said "It's fine now", wouldn't I? But when you see you're not moving forward and she couldn't care less if you turn up or not, then no, there can't be much trust there. (Girona, male patient)*

B. Factors influencing relational continuity

Category	Quotations	
	**Primary care doctor**	**Secondary care doctor**
**Related to the health system**		
**Gatekeeper functions** lead to consistency of primary care doctors and frequent visits, necessary to develop an ongoing relationship	*Well of course I can't talk about a specialist like I can about my GP, because I only go to the specialist every now and then. With my GP we deal with every little thing, illnesses, colds, this and that, I see him a lot. In one month you might see him three or four times and then a whole year goes by without going, but the relationship is closer. (Girona, male patient)*<br>*For asking questions and all that, yes, it's my GP (who I confide in most), I mean, she's the one I have most contact with, the one I visit the most every year. If I see each specialist maybe two or three times a year at the Hospital del Mar, I'll see my GP twice as often as that. (Barcelona, male patient)*	
**Related to the health services organizations**		
**Appointment-making system** promotes being seen by the same doctor	*It's always her (GP), always the same one (…) Yeah, because when you ask for an appointment, for example, I say "I want an appointment for Friday", "Ah, no, the doctor isn't here on Friday, she's here Monday", "Ah, right, well maybe Monday then". So no one else can see you instead, because if she isn't there on the day you ask for, then you have to get an appointment for another day when she is there. (Baix Empordà, male patient)*	*Yes, always the same (pulmonologist). Since I've been having treatment, it's always been the same one (…) I've never had to visit a substitute when she's off on holiday. Instead, they book my appointment for when she comes back or before she goes. So I've never had the experience of seeing someone else. (Girona, male patient)*

**Reorganization of patient lists** results in the assignation to a new doctor	*They've changed ours (GP), not because we requested it of course! But for whatever reason, maybe because there are some doctors who have more patients than others, I think that's probably the reason, don't you? That they have to share out the work a bit so it's not the case that one doctor has lots of patients whilst others have a few. I imagine that's what it's all about, don't you? Just to even out everyone's workload a bit. (Baix Empordà, female patient)*	*My digestive specialist is Doctor (name), but last year or the year before I was seen by this one (…) Maybe because they've got lots (of patients) and they can't attend to everyone, so of course they share the work out between them. (Baix Empordà, female patient)*
**Small size of the primary care** centre limits possibility to change the doctor	*No (changes), because in San Antoni, there were only two doctors (GPs). A lot of people, some had the female doctor and others had the male doctor. (Baix Empordà, male patient)*	*The same one (orthopaedic surgeon), yes (…) that's all there is, I don't get to choose, that's what there is here, in the (primary care) health centre, it's all there is. (Barcelona, male patient)*
**Sufficient consultation time** favours the development of an ongoing relationship	*(The relationship) good, good, yes. (The GP) is nice and in spite of the pressure they're under – it's a lot of patients to see in very little time – she's up to it. I mean, if you need more time, she gives you more. So if my appointment's at 10, for example, I know I won't be out until at least 10.30. She takes her time when someone needs it, and you're in there for longer. (Barcelona, male patient)*	*The ones I have a closer relationship with are the doctor from pulmonology, and the one who takes your blood pressure. Every time I go that's who I spend most time with, I always see her in the surgery. Both the nurse and the doctor. The ones who see me the most. (Barcelona, male patient)*
**Related to the doctors**		
**Doctor's adequate medical practice** favours the development of an ongoing relationship	*My current doctor, who is Doctor (name), really good (…) because always, whenever I've come in she's attended to me well (…) she found, looking at the tests, that I've got a bit of a thyroid problem, so, well yes, I appreciate that too (…) she started the whole process and they gave me an ultrasound here, at the specialist, yeah? Well, she detected it because she's good at her job. (Girona, female patient)*	*I'd rather it was the same one (cardiologist) because I have a lot of trust in him (…) And up till now he hasn't disappointed me, with all the stuff he's told me, so yes, good. And I really appreciate that. (Barcelona, male patient)*

**Effective patient-doctor communication** favours the development of an ongoing relationship	*It's a really nice relationship that I've got with my doctors, actually. In fact with Doctor (name of the GP), we talk about plants, because she really likes flowers and I do too, and we talk about plants (…) She's like a lifelong friend, it's a really good relationship, and if I have any questions I ask her, in complete confidence. (Girona, female patient)*	*I get on pretty well with them (pulmonologist and her nurse…) because of the way they communicate with their patients, or with me at least. They always try to find out, like, how you're feeling, how the past few months have been for you. They talk to you in a friendly way and they care a lot about your state of health. (Barcelona, male patient)*
**Physician's commitment to patient care** favours the development of an ongoing relationship	*I'm very happy with the Gotic (primary) health centre, with the doctors I have (…) they're people we're really happy with, because they go beyond the call of duty, shall we say (…) I don't know, they've even called me at home to see how I am, and, well, that's something to be really grateful for. (Barcelona, female patient)*	*There are specialists like the one we had before, an ophthalmologist, that, well…I realise that he was a good professional but he didn't know how to treat people. Why? Because I'm sure he had to see a lot of people in a very short space of time, he only had, I don't know, ten minutes to see them, and that meant that (…) he would treat the patient almost like just another number, like another part on the assembly line, and that does hurt, because at the end of the day we're all human beings. (Barcelona, male patient)*
**Physician's positive traits** favours the development of an ongoing relationship	*I'm really happy with her (…) it's that personal touch you get with her (…) and every time you go she welcomes you with a smile, I don't know, she's the kind of person that makes the patient feel like they've been given a dose of medicine without even having received any treatment. (Girona, male patient)*	*There is real harmony, yes indeed (with the urologist), we get on well. I'm a very open person, she's very open and really nice. (Barcelona, male patient)*
