# Supplementary material for: Relational continuity with primary and secondary care doctors: a qualitative study of perceptions of users of the Catalan national health system
Source: BMC Health Serv Res. 2018 Apr 10;18:257. doi: 10.1186/s12913-018-3042-9 (PMC5891958; doi:10.1186/s12913-018-3042-9)
Supplement: Supplementary file 2 — Original Spanish version of the quotations. (PDF 291 kb) [file 12913_2018_3042_MOESM2_ESM.pdf]

Supplementary file 2 – Original Spanish language version of the quotations of relational continuity with primary and secondary care physicians

A. Patients' perceptions of relational continuity

Category	Quotations	
	**Primary care doctor**	**Secondary care doctor**
Consistency of doctors over time	*Desde que se jubiló el otro (médico de cabecera) que tenía, pues, no sé, mejor hace 15 o 20 años ya que estoy con ella. Es mucho, mucho tiempo (…) Siempre la misma. (Baix Empordà, male patient)*<br>*Alguna vez la muchacha pues ha tenido que ir a algún sitio, y he quedado con otro (…) ha sido muy pocas veces (…) una vez o dos. (Girona, male patient)*<br>*Con esta doctora perfecto. Una vez coincidió que ella estaba de vacaciones o una vez me parece que estaba enferma pero es normal, todo nos ponemos enfermos, y todos hacemos vacaciones. (Baix Empordà, female patient)*<br>*¿Médicos suplentes? No que me encontrado. (…) En el CAP normalmente siempre me visita mi médica (…) a no ser que ella, pues ya te digo, esté de baja. (Barcelona, male patient)*	*A veces pues te ponen a un día la visita y ese día el médico (especialista) pues no puede por mucho que sé, por alguna razón, la que sea y te ponen otro, te ponen el suplente (…) no (molesta), no, al contrario, estaba bien empapado de lo que me pasaba, sabía perfectamente y ningún problema, por eso que no noté la diferencia. (Baix Empordà, male patient)*<br>*La oncóloga que me la han cambiado una vez sólo (…) Claro, como a ver en todos los sitios hay movilidad, ¿no? La oncología, sí, hubo un cambio pero incluso la veía en el otro despacho porque se cambió de especialidad (…) se las veía muy ¿sabes? como muy coordinados para hacer el traspaso (…) Quiero decir coordinados porque una se fue y entonces la otra la sustituyó. "Sí, sí, me ha dicho tal." (…) no ha sido un problema. (Barcelona, female patient)*<br>*(El nuevo neurólogo) estupenda (…) Me cambió el medicamento. Me dijo que llevaba demasiado tiempo tomando el medicamento para la migraña y que me podía dañar al… tanto al corazón, como a los riñones y a la vista. Me lo cambió y bueno, ahora estoy probando uno nuevo a ver qué tal aguanto. (Barcelona, male patient)*<br>*A veces pues te ponen a un día la visita y ese día*

		el médico pues no puede por mucho que sé, por alguna razón (…) no (molesta), no, al contrario, estaba bien empapado de lo que me pasaba, sabía perfectamente y ningún problema, por eso que no noté la diferencia (…) se había leído mi historial (Baix Empordà, female patient).
Developed ongoing relationship based on		
a) mutual accumulated knowledge	*A mí, me conoce yo creo que mi madre me conocía bien, pero yo creo que ella…ella me conoce mucho más. Hasta ha llegado a decirme 'tú eres una persona que se queja muy poco'. Y es verdad. Me da hasta rabia, digo '¡es que sabe más de mí que yo mismo!' (… El sustituto) no lo sabe personalmente o memorizado como ella (la médico de cabecera), por ejemplo como me conoce a mí como persona. (Girona, male patient)*<br>*Si ella (la médica de cabecera) hace vacaciones o algo, pues sí, hay suplentes, claro (…) simplemente hace la vista y marchando porque es diferente con ella, ¿lo entiende? el suplente no sabe bien bien lo que te pasa aunque vea allí en el ordenador, que este señor tiene azúcar o tensión alta o lo que sea, pero no es lo mismo, ¿no? La doctora ya lo sabe sin mirar nada. (Baix Empordà, male patient)*<br>*Llevo unos casi 10 años (con la médica de cabecera) (…) el médico te conoce a ti y casi sin mirar el historial pues sabe de qué va. (Barcelona, male patient)*	*Por lo de la lumbalgia, me ha visto dos o tres veces y sí que me conoce, ahora a lo mejor si no vuelvo de aquí a 6 meses, o sea a lo mejor me tiene visto, pero no, es normal. Una vez si vas mucho, es como todo el mundo, si tú ves a una persona mucho al final la conoces pero si le ves de cuando en cuando te suena y ya está. (Barcelona, male patient)*<br>*(El traumatólogo y el médico de cabecera) son médicos que hace años que los tenemos. No hace cuatro años, ni ocho. Hace muchos años. Y nos conocemos bien. Y ellos saben cómo somos nosotros y nosotros más o menos cómo actúan ellos. Estamos contentos con ellos. (Girona, female patient)*

b) personal trust in the doctor	*El de cabecera que es el que digamos que tengo la más confianza, porque siempre ha sido el mismo hoy por hoy (…) Hay una confianza porque son muchos años, si llevo con el más de 30 años. Yo prefiero aquí contigo y tú eres como una persona de la familia que tengo una confianza contigo que puedo hablar abiertamente. (Girona, male patient)*	*Con el cardiólogo (…) tengo mucha confianza y ya llevo muchos años y siempre ha sido el que me ha visitado allí. Me inspira, me inspira confianza y veo que con lo que me dice, comprendo y yo le entiendo y veo que tiene razón. Yo tengo mucha confianza, y hasta ahora no me ha defraudado, con todo lo que me ha dicho, o sea que bien. (Barcelona, male patient)*
	(En urgencias) no se desenvuelven como deberían desenvolverse en las emergencias, era con cada uno de los pacientes que llegan. (El médico de cabecera) es mejor porque ya hay un grado de confianza con ellos, ¿no? Tienes más libertad de hablar y de decirle que con los médicos que tratan así esporádicamente, que no tienes mucha relación con ellos. (Girona, male patient)	*Algunas veces no sé si era el estrés pero (la fisioterapeuta) era un poco borde. (…) Bueno, confianza, te digo que no, porque si hubiese habido confianza, creo que hubiese seguido el tratamiento hasta que ella me dijera "Está bien", ¿no? Pero al ver que no pasas de lo mismo y que le da igual si vienes o si no, entonces no, no puede haber confianza. (Girona, male patient)*
	A cualquier persona puedes tenerle confianza pero si es la que te ha llevado siempre, es la tuya, lo que tú confías con aquella persona. Yo he visto a mucha gente en la consulta levantarse e irse, porque sabían que no estaba esta doctora. (Baix Empordà, female patient)	

Translated quotations of the results section in the article are highlighted in blue colour.

B. Factors influencing relational continuity

Category	Quotations	
	Primary care doctor	Secondary care doctor
**Related to the health system**		
**Gatekeeper functions** leads to consistency of primary care doctors and frequent visits, necessary to develop an ongoing relationship	*Claro, un especialista yo no puedo hablar como de mi médico de cabecera, porque yo con el especialista voy cuando voy. Mi médico de cabecera es el trato que tenemos de cada cosa, de enfermedades, de costipados, de esto, de aquello, le veo muchas veces. A lo mejor un mes lo ves tres o cuatro veces y luego te tiras un año que no lo ves, pero el trato es más cercano. (Girona, male patient)*<br>*A la hora de preguntar las cosas y demás sí, es la doctora de cabecera (que tiene más confianza), a ver, es con la que más trato, es con la que más veces voy al año. Si a cada especialista veo, por ejemplo, pues dos o tres veces al año en el Hospital del Mar, a la doctora de cabecera la veo el doble. (Barcelona, male patient)*<br>*A lo mejor voy con una de la familia, la acompaño yo, la veo mucho, entonces es diferente (…) el trato es más cercano. (Girona, male patient)*	
**Related to the health services organizations**		
**Appointment making system** promotes being seen by the same doctor	*Siempre es ella (médica de cabecera), siempre la misma (…) Si, porque cuando vas a pedir turno, eh...yo por ejemplo le digo "quiero un turno para el viernes", "ah, no, el viernes la doctora no está, ella está el lunes", "ah vale, entonces a lo mejor el lunes". Entonces, no te puede tomar otra, porque si ese día que pediste no está pues tienes que*	*Sí, siempre la misma (neumóloga). Desde que me ha estado tratando, ella es la misma (…) No me ha tocado el cambio de llegar cuando ella está de vacaciones. Si no, me dejan la cita cuando ella viene o antes. Entonces no he tenido ninguna experiencia con otro. (Girona, male patient, 42)*<br>*El especialista del aparato digestivo (…) ha sido la*

	pedir para el día que esté. (Baix Empordà, male patient)	*misma, una digestóloga (…) con el especialista siempre ha sido ella (…) porque si ella tiene esta fecha y no puede estar luego me avisan y me dan otra fecha, pero siempre es la misma. (Girona, female patient)*
**Re-organization of patient lists** results in the assignation to a new doctor	*Nos lo han cambiado (de cabecera) no porque nosotros no lo hayamos pedido eh!, porque por el motivo que sea a lo mejor porque hay médicos que tienen más pacientes que otros, y creo que debería ir la cosa por ahí ¿no? Que también deben repartir un poco el trabajo para que no un médico tenga muchos pacientes y otros menos. Yo creo que cada cosa debe ir por ahí ¿no? Si no para equiparar un poco el trabajo de cada uno. (Baix Empordà, female patient)*<br>*(…) se ve que estaban organizando un poco los médicos con los pacientes y yo no sé, supongo sería porque hay muchos inmigrantes, los marroquíes que no se encuentran cómodos con los médicos. (…) como la doctora tenía demasiados clientes, bueno pacientes y supongo que tendrían que reestructurar un poco. (Baix Empordà, male patient)*	*Yo mi médico digestólogo es el doctor (nombre) pero ya el año pasado o hace dos años me visitó esté (…) A lo mejor porque tienen muchos y no pueden atender a todos, y claro ya se reparte la faena. (Baix Empordà, female patient)*
**Small size of the primary care** centre limits possibility to change the doctor	*No (hay cambios) es que en San Antoni, solo habían dos médicos. Mucha gente, unos tenían a la doctora y otros tenían el doctor. (Baix Empordà, male patient)*<br>*Nunca lo pensé (cambiar el médico de cabecera), la verdad, nunca lo pensé porque allí en Calonge solamente creo que tienen dos médicos. Son dos médicos, de eso al pasar al otro. (Baix Empordà, male patient)*	*El mismo (traumatólogo) si (…) esto es lo que hay, yo no puedo elegir, es lo que hay aquí en el centro, en el CAP, es lo que hay. (Barcelona, male patient)*

**Sufficient consultation time** favours the development of an ongoing relationship	*(La relación) bien, bien, sí. (La médica de cabecera) es simpática y a pesar de la presión que tienen - que hay muchos pacientes en poco tiempo - está por la labor. Es decir, si necesitas más tiempo está más tiempo. De manera si me toca a las 10, por decir algo, ya sé que por lo menos como muy pronto hasta las 10.30 no voy a salir. Pues se entretiene cuando alguien necesita, y estás más tiempo. (Barcelona, male patient)*	*Con los que tengo mejor relación es con la doctora de neumología, con la de la tensión. Cada vez que voy es con quien más tiempo estoy, que la veo en la sala. Tanto con la enfermera como con la doctora. Los que más me miran. (Barcelona, male patient)*<br>*En el caso de aquí del Hospital del Mar (…) pues a lo mejor me paso media hora comentando (con el neurólogo y el nefrólogo…) Al menos parece que se interesan más por los pacientes. En el Hospital Clínico como se suele decir, "van a saco". Entras, bla bla bla, va, fuera. Supongo que será pues que como es más grande, está más centralizado pues tienen mucha más cantidad de gente. (Barcelona, male patient).*
**Related to the doctors**		
**Doctor's adequate medical practice** favours the development of an ongoing relationship	*Lo que es mi doctora actual, que es la doctora (nombre), muy bien (…) porque siempre, pues cuando he venido me ha atendido correctamente (…) ella encontró, viendo los análisis, que tenía algo de tiroides, entonces, bueno, pues eso también lo valoro (…) inició todo el proceso y me han hecho una ecografía de aquí, ya en el especialista, ¿no? bueno, lo detectó ella porque trabaja bien. (Girona, female patient)*	*Prefiero que sea el mismo (cardiólogo) que tengo mucha confianza (…) Y hasta ahora no me ha defraudado, con todo lo que me ha dicho, o sea que bien. Eso también vale mucho. (Barcelona, male patient)*
**Effective patient-doctor communication** favours the development of an ongoing relationship	*Es una relación muy bonita yo con la que tengo con mis médicos, eh. Incluso con la doctora (nombre) hablamos de plantas, porque a ella le gusta mucho las flores y a mí también (…) Es como amigo de siempre, muy buena relación, y si tengo alguna duda se la pregunto, con mucha confianza. (Girona, female patient)*<br>*La última vez que estuvimos y estuvimos hablando*	*Me entiendo bastante bien con ellos (neumóloga y su enfermera…) por la forma de comunicarse con los pacientes, al menos conmigo. Siempre pues tratar de averiguar pues cómo te encuentras, cómo has pasado estos últimos meses. La comunicación es muy cordial y se interesan mucho por el estado de salud. (Barcelona, male patient)*

	poco rato, pero estuvimos hablando de los recortes y tal. (…) La barrera médico-paciente que es importante y tal pues se bajó un poco. Se hizo más próxima y supongo que también yo me hice más próximo a ella, la relación fue más humana, por decirlo de alguna manera. (Barcelona, male patient)	
**Physician's commitment to patient care** favours the development of an ongoing relationship	*Yo estoy encantada con el CAP del Gótic, con los médicos que tengo (…) son personas que estamos muy contentas, porque su dedicación va más allá de lo estrictamente oficial, digamos (…) incluso yo que sé, me han llegado a llamar a casa a ver cómo estás, esto es, no sé, muy de agradecer. (Barcelona, female patient)*<br>*Tengo bastante confianza en ella, sí. Además creo que se esfuerza (…) se interesa por el paciente, y eso me parece importante. (Barcelona, female patient)*	*Hay especialistas como teníamos un oftalmólogo antes, pues que yo lo reconozco como un buen profesional pero que no sabía tratar a la gente. ¿Por qué? Porque seguramente debía tratar a mucha gente en muy poco tiempo, solamente tenía pues no sé diez minutos para tratarlos, y eso hace que (…) trate al paciente casi como un número, casi como un elemento más de la cadena de montaje, y a mí esto sí que me duele mucho porque al final somos personas. (Barcelona, male patient)*
**Physician's positive traits** favours the development of an ongoing relationship	*Yo estoy contentísimo de ella (…) es el trato personal que tienes con ella (…) y siempre que vas te sonríe, no sé, es una persona que el enfermo parece que está dando una medicina sin haber hecho ningún tratamiento. (Girona, male patient)*<br>*Es un familiar, muy campechano, muy tranquilo. Es agradable. Te da confianza. (Girona, female patient)*	*Hay buena harmonía vaya (con el urólogo), nos tratamos bien. Yo soy una persona muy abierta, ella es muy abierta y muy simpática (Barcelona, male patient)*

Translated quotations of the results section in the article are highlighted in blue colour.
